# Some Landmarks in Flanders

**Published:** 1919-10-11

**Authors:** 


### SOME LANDMARKS IN FLANDERS.

### From the Notebooks of a Medical Officer.

During the whole of the war, practically, a considerable part of the British Forces on the Western Front were holding that all-important piece of the old trench line which protected Calais, Dunkirk, and Boulogne, and ran, roughly speaking, from the Yser to Bethune. The intermediate country, between the battle zone and the sea, contains no large towns, but it is nevertheless densely populated by an agricultural people, aggregated in hundreds of small villages and thousands of single homesteads; and for this reason it could, and did, afford shelter to the large armies which had to be kept in reserve in that district. Medical units in particular had ample opportunities during slack times of exploring this segment of old Flanders, the greater part of which was filched piecemeal from the Spanish rule by the French during the seventeenth century, though some of it is still united to the Belgian crown.

In this piece of territory, for the most part very flat in character, every town and almost every village had been the scene of the exploits of the British Army during the six score years which ended in 1815. Cassel, perched on the summit of a very steep hill some 600 feet high, is famous as the place where the Duke of York once "marched his army up the hill and then he marched them down again." No one who knows the heartbreaking pitch of that hill can wonder that the troops "swore terribly in Flanders," choked as they were by the high stocks of those days. A monument at the top of the hill recalls various battles close to the town, from the eleventh century onwards, in which the British Army was not engaged. One of these, commemorated also by a memorial stone on the actual battlefield near the village of Zuytpeene, gave Flanders to France in the days of Louis XIV.; the Cassel monument alludes to a prince of the Royal house as the conqueror, but some sceptical Frenchman has graved on the stone a mark of interrogation after the word "vainqueur." Not far from Zuytpeene is one of the oldest churches of the vicinity, that of Wemaers Cappel, which has some fifteenth-century monuments in the nave. But the most curious object in this church hangs on the wall in a gold frame near an altar dedicated to St. Blasius. An inscription records that on that saint's feast day, in about 1750, the vicar of a neighbouring church was eating a carp when part of its backbone "went down the wrong way"—in fact it seems to have lodged in his larynx. "Il se sentit mourir," says the chronicler, as indeed he no doubt did. In this extremity he called upon St. Blasius to deliver him from the plight in which he found himself: for it was in special honour of the saint that he was eating the carp. No sooner had he prayed thus to Blasius than he coughed out the bone, and was soon all right. In gratitude he had the bone mounted in gold and sent to the nearest church in which St. Blasius had an altar: and there it is to this day, to confute the sceptic.

Ypres, Menin, Furnes, Dixmude, Ostend, Dunkirk, Lille, are names that occur over and over again in the itineraries of Marlborough's armies and of later eighteenth-century British Expeditionary Forces. The last named was taken by the Allies under Marlborough in 1708, in spite of the bribe of two million crowns offered by the French king to the Duke if he would raise the siege. It was alleged against Marlborough by his enemies that he purposely left an important convoy from Ostend insufficiently escorted, so as to have an excuse to raise the siege if the convoy were cut off: an intention frustrated by the courage and address of Brigadier-General Webb and his troops, who routed the French force—six times their own number—detached to intercept the convoy. This was at Wynendael, not far from Bruges, and north of the areas that the British armies have occupied during this war.

St. Omer, again, is well known to hundreds of thousands of British soldiers: yet how many have seen in the apse of the Cathedral the burial inscriptions of two grandchildren of that Baldwin, Count of Flanders, under whom Hereward the Wake, the last of the Anglo-Saxons, served at St. Omer? Here in St. Omer Hereward met Torfrida, who became his wife. The Cathedral, amongst many other interesting relics, contains a large group of statuary presented by Charles V. of Spain during the time that the town was part of his dominions. In the South transept is a curious and somewhat crude statue of the seated Madonna, seemingly carved in wood. It is kept at the top of a shrine so lofty that from the floor-level it is difficult to be sure of the detail. Photographs are, however, on sale, and from them one can make out that the physiognomy is indubitably and markedly Tartar. So pronounced is this that it is perhaps questionable whether the statue, which has been in this Cathedral six or seven hundred years, was originally carved to represent the Virgin at all. Once a year the statue is carried in procession round the church, at which time sick pilgrims come from long distances round in the hope of being cured of their diseases. The pillars and walls of the South transept are covered with inscriptions, dating from the fourteenth century down to the twentieth, recording many such therapeutic miracles. St. Omer shows more traces of the Spanish occupation than most other towns in Flanders: two or three of the main streets are full of old houses in the Hispano-Flemish style. The forest of Clair Marais, just outside the town, is said to have furnished the oak timber for Julius Cæsar's ships, which were launched at Watten, six miles away; the latter village was in 1917 and 1918 seldom without a brigade or division "in rest." The Portus Itius, whence Cæsar sailed on his expedition to Britain, has not been identified with certainty: the best authorities seem to think it was Wissant, near Ambleteuse, between Boulogne and Calais.

**Some Landmarks in Flanders**—(*continued*).

Bergues, nearer Dunkirk, has one very fine Spanish house of the late sixteenth century, as well as massive old ramparts surrounded by a moat, and a good belfry. As far as the British Army is concerned, it is chiefly interesting for the record on the wall of the barracks of a successful sortie by the garrison in the eighteenth century against a British Army retreating towards the coast from inland. Esquelbecq, in the same district, has a fine red-brick château built in 1606 and moated by the Yser, here a jumpable streamlet. Said to contain eighty bedrooms, this beautiful building is cut out for the headquarters of armies. Marlborough billeted troops there: and it is said that two at least of these identical British regiments have been billeted at Esquelbecq again during this war. The fine old inn near the château gates, dated 1617, is kept by a middle-aged landlord who proudly boasts that after Waterloo his grandfather had one of Wellington's brigadiers billeted at the inn; the family tradition seems not to run as far back as Marlborough's time. The two Wolfes, he who afterwards fell at Quebec, and a brother, soldiered in Belgium together as ensigns. In two extant letters home the younger Wolfe refers to "ammunition bread" as far preferable to that obtainable from the countryfolk: a phrase which shows that even then the British commissariat had its strong points, and also that the current army slang of "ammunition" used as an adjective to denote "supplied by Government," is as old, at the least, as 1743.

To go a little further afield, Tournai, on the Scheldt, is full of old associations. Besides the conquering tour of Henry VIII., described last year in the *Times*, it is also noteworthy for its propinquity to Fontenoy, where in 1745 a British Army under Cumberland failed, in spite of prodigies of valour, to relieve Tournai, then besieged by Marshal Saxe. Fontenoy is some three miles or so south of Tournai, on the right (east) bank of the Scheldt. The story of the battle reveals very little generalship on the part of the British commander, who sent his troops to a frontal attack on an entrenched position held by superior numbers of veteran troops, well supplied with artillery. Even then the attack might have succeeded, thanks to the valour of the regimental officers and men, if the brigadiers had all understood what it was they were expected to do. An Irish brigade contributed materially to the French victory. Pont à Chin is quite close to the town: in May 1794 the French captured it from us five times in one day, only to be five times driven out by our troops: though not an affair of great strategic importance, there were 9,000 casualties in this bitterly contested engagement. Tournai has the finest Cathedral in these parts: all the nave and both transepts are magnificent Norman work of 1010 and thereabouts; the choir is rather ordinary fifteenth-century Gothic; there is a very fine marble choir screen of the twelfth century; and in the chapter house some splendid Arras tapestry of the thirteenth, fourteenth, and fifteenth centuries.

Lille, too, which during the war was behind the German lines, is a very old city, though only a few streets of the old part remain. Wandering one day along one of these, the writer noticed that it was called the Rue d'Angleterre, and wondered whether it had received this name after the surrender to Marlborough. A mural inscription at the end of the street, however, showed that the name has a much greater antiquity than this; for it recorded that "Saint Thomas of Canterbury" had lodged there for some time. This, of course, was Thomas à Becket, as we are used to calling him; naturally he would stay in an English priory or other religious foundation, after which, no doubt, the street was named. Both Dunkirk and St. Omer, by the way, have English churches; that at the latter town is described in old maps of the town in the seventeenth and eighteenth centuries as Jesuit property, and it very possibly is so still. The Dunkirk English Church is also old, and may date from the time when we held this seaport, which was sold by Charles II. to the French when Parliament did not vote him enough for his spendthrift habits. Not far from Lille is Pont à Bouvines, where King John of England was defeated by the French: it was the consequent disintegration of his army that enabled the barons, on his return, to dictate to him at Runnymede, and compelled him to sign Magna Charta. In the same vicinity are Roubaix and Mouvaux, large flourishing industrial towns before the war, and destined to be so once more. Here in 1794 fierce fighting took place between our forces, including the Guards, and the French: at first we were successful, but the French were reinforced and a day or two later we lost all the prisoners we had taken and some guns as well. Generals Fox and Abercrombie were the brigadiers on our side, under the command of the same Duke of York already mentioned in connection with Cassel.

